# Burden of critically ill patients with influenza in a French catchment population

**DOI:** 10.1038/s41598-021-89912-y

**Published:** 2021-05-18

**Authors:** Romain Hernu, Marie Simon, Thomas Baudry, Jean-Sébastien Casalegno, Bruno Lina, Martin Cour, Laurent Argaud, the “Flu in Lyon ICUs” Study Group, Frederic Aubrun, Claude Guérin, Bernard Allaouchiche, Dominique Robert, Julien Bohé, Marc Puidupin, Jacques Manchon, Lionel Liron

**Affiliations:** 1 Service de Médecine Intensive-Réanimation, Hospices Civils de Lyon, Hôpital Edouard Herriot, 5, place d’Arsonval, 69437 Lyon Cedex 03, 69003 Lyon, France; 2 CNR Des Virus Des Infections Respiratoires, Hospices Civils de Lyon, Institut Des Agents Infectieux, 69004 Lyon, France; 3 Faculté de médecine Lyon-Est, Université de Lyon, Université Lyon 1, 69008 Lyon, France; 4 Hospices Civils de Lyon, Hôpital de La Croix-Rousse, Lyon, France; 5 Hospices Civils de Lyon, Hôpital Edouard Herriot, Lyon, France; 6 Hospices Civils de Lyon, Hôpital Lyon-Sud, Pierre-Bénite, France; 7 Hôpital Desgenettes, Lyon, France; 8 Hôpital Privé Saint Joseph Saint-Luc, Lyon, France; 9 Hôpital Privé Tonkin, Villeurbanne, France

**Keywords:** Diseases, Infectious diseases, Influenza virus

## Abstract

Despite the particular focus given to influenza since the 2009 influenza A(H1N1) pandemic, true burden of influenza-associated critical illness remains poorly known. The aim of this study was to identify factors influencing influenza burden imposed on intensive care units (ICUs) in a catchment population during recent influenza seasons. From 2008 to 2013, all adult patients admitted with a laboratory-confirmed influenza infection to one of the ICUs in the catchment area were prospectively included. A total of 201 patients (mean age: 63 ± 16, sex-ratio: 1.1) were included. The influenza-related ICU-bed occupancy rate averaged 4.3% over the five influenza seasons, with the highest mean occupancy rate (16.9%) observed during the 2012 winter. In-hospital mortality for the whole cohort was 26%. Influenza A(H1N1)pdm infections (pdm in the mentioned nomenclature refers to Pandemic Disease Mexico 2009), encountered in 51% of cases, were significantly associated with neither longer length of stay nor higher mortality (ICU and hospital) when compared to infections with other virus subtypes. SOFA score (OR, 1.12; 95% CI, 1.04–1.29) was the only independent factor significantly associated with a prolonged hospitalization. These results highlight both the frequency and the severity of influenza-associated critical illness, leading to a sustained activity in ICUs. Severity of the disease, but not A(H1N1)pdm virus, appears to be a major determinant of ICU burden related to influenza.

## Introduction

Influenza, common respiratory viral infection, is estimated to result in about 290,000 to 650,000 deaths each year worldwide^1^. Young people, elderly or those with an underlying medical condition are more likely to develop serious forms of the disease^2^. Influenza infections cause exacerbations of chronic diseases as well as specific complications such as severe viral pneumonia, leading to substantial increase in hospital admission and deaths^2–4^.

In addition to annual winter epidemics, influenza viruses also cause recurring and unpredictable pandemics^2,5^. The 2009 A(H1N1) pandemic specifically affected young populations without major comorbidity and therefore attracted much attention from the public and policy-makers^6^. Abundant medical literature has borne witness to this newsworthy 2009 A(H1N1) pandemic^6–13^. As a consequence, considerable human, economic and scientific resources were mobilized^13^. Bed-occupancy rate was a sensitive topic, including in the intensive care units (ICUs) which were in the front line for the management of severe influenza-infected patients. However, since the end of the 2009 A(H1N1) pandemic, reports on the impact of influenza in ICUs during subsequent outbreaks remain scarce.

Here, we present a multicenter prospective study of critically ill influenza-infected patients in a catchment population between 2008 and 2013, i.e. before, during and after the 2009 A(H1N1) pandemic. Thus, the aim of the present work was to report the reality of influenza-associated critical illness and to identify factors associated with influenza burden in ICUs.

## Methods

The study was performed in compliance with the ethical standards of the Declaration of Helsinki and according to French laws. The ethics committee, *Comité de Protection des Personnes Sud-Est II*, approved this multicentric prospective non-interventional study (Reference number: CAL N° 2012–024). This Institutional Review board waived the need for informed consent given the observational nature of the study.

### Study design

This study was conducted from December 2008 to April 2013 in the large Lyon catchment area in France (534 km^2^; 1.3 million inhabitants). Unspecialized medical and surgical ICUs of the 12 centers (154 beds) that usually receive influenza patients in this area participated in the study. Five periods were defined based on the five influenza winter epidemics of the Northern hemisphere, according to the French surveillance network (Institut de Veille Sanitaire) in the Auvergne Rhône-Alpes region. Prospective inclusions started from the 2009 A(H1N1) pandemic while data from winter 2008 were retrospectively collected.

All adult patients (≥ 18 years old) admitted to the ICU with influenza infection were included. Virological diagnoses of influenza were made using specific Real-Time Polymerase Chain Reaction (RT-PCR) assays performed on either nasopharyngeal swab specimens (for non-intubated patients) or bronchoalveolar lavage samples (for intubated patients). RT-PCR was also used for virus subtyping. By default, serological analysis using hemagglutination inhibition and complement fixation tests could be performed to confirm influenza infection.

### Data collection

For each patient, the following characteristics were recorded: demographics, vaccination for influenza (within the current year), life-expectancy using the McCabe and Jackson scale ^14^. Severe obesity was defined as a BMI greater than 35 kg/m^2^. The time course of the acute illness and the reason for hospitalization were both collected upon ICU admission. Severity of illness was assessed using both the Simplified Acute Physiology Score II (SAPS II) and the highest Sequential Organ Failure Assessment (SOFA) score during ICU stay^15,16^. The SOFA score at ICU admission was also used to compare patients’ characteristics according to in-hospital length of stay. Organ supports were also recorded; diagnosis of Acute Respiratory Distress Syndrome (ARDS) was made in accordance with the 1994 American-European consensus-conference^17^.

Mortality was evaluated at day-28, and at both ICU and hospital discharge. Lengths of stay in the ICU and in-hospital were also collected. The ICU bed occupancy rate by Influenza-infected patients was computed as the number of beds occupied by influenza-infected patients divided by the total number of beds available in the participating centers.

### Statistical analysis

Values are expressed as mean ± standard deviation (SD) or number (%), as appropriate. Univariate comparisons were performed using Mann–Whitney U test for continuous data and Chi-2 or Fisher’s exact tests for categorical data, as appropriate. The independent contribution of parameters available at time of admission and during ICU stay to the in-hospital length of stay was analyzed using a backward stepwise multivariate analysis in a logistic regression model. In-hospital length of stay was dichotomized according to the median value. Following univariate analysis, all variables with *p* ≤ 0.10, as well as age, sex and A(H1N1)pdm viral subtype, were included in the logistic regression model. Potentially confounding factors were eliminated if *p* values were > 0.10. Odds ratios (OR) were estimated with 95% confidence intervals (95% CI).

Statistical calculations were performed using Medcalc Statistical Software version 12.1.2 for windows (MedCalc Software bvba, Ostend, Belgium). A *p* value < 0.05 was considered statistically significant.

## Results

During the study period, 201 patients met the inclusion criteria. Table 1 shows the characteristics of the patients for the five influenza seasons. In this population, 124 (62%) patients were over 60 years old. The whole cohort included 29 (14%) obese patients, two (1.0%) health care workers and two (1.0%) pregnant women. Patients were significantly younger in 2009 than those hospitalized during other influenza seasons (54 ± 14 versus 65 ± 16 years old, respectively, *p* < 0.001). The proportion of patients with ARDS was constant over time, with resort to Extra-Corporeal Membrane oxygenation (ECMO) during 2009 and 2010 winters only (Table 1). Also, no significant difference in the severity of patients’ illness was observed over the study period.

**Table 1:** Patients’ characteristics.

	2008 n = 5	2009 n = 40	2010 n = 41	2011 n = 40	2012 n = 75	Total n = 201
***Epidemiology***						
Age (years)	67 ± 25	54 ± 14	57 ± 15	72 ± 13*	65 ± 15*	63 ± 16
Sex-ratio	0*	1.2	2.2	1.1	0.9	1.1
Influenza vaccination	0	4 (10)	5 (12)	10 (25)	6 (8.0)	25 (12)
***Comorbidities***						
None	2 (40)	9 (23)	4 (10)	2 (5.0)*	2 (2.7)*	19 (9.5)
*Type*						
Chronic pulmonary disease	0	10 (25)	16 (39)	19 (48)	26 (35)	71 (35)
Chronic heart disease	1 (20)	7 (18)	6 (15)	8 (20)	20 (27)	42 (21)
Renal insufficiency	1 (20)	6 (15)	7 (17)	2 (5.0)	6 (8.0)	22 (11)
Immune depression	2 (40)	13 (33)	12 (29)	4 (10)*	16 (21)	47 (23)
Pregnancy	0	0	1 (2.4)	0	1 (1.3)	2 (1.0)
Severe obesity	0	6 (15)	6 (15)	5 (13)	12 (17)	29 (14)
*Life expectancy*						
None or non-fatal	2 (40)	25 (63)	26 (63)	30 (75)	42 (56)	125 (62)
Fatal within 5 years	2 (40)	12 (30)	14 (34)	9 (23)	28 (37)	65 (32)
Fatal within 1 year	1 (20)	3 (7.5)	1 (2.4)	1 (2.5)	5 (6.7)	11 (5.5)
***ICU stay***						
Symptom duration before ICU (days)	8.8 ± 6.5	4.5 ± 3.2	5.6 ± 4.5	3.9 ± 4.0	5.6 ± 6.1	5.1 ± 5.0
*Main reason for admission*						
Respiratory distress	5 (100)	38 (95)	35 (85)	33 (83)	63 (84)	174 (87)
Shock	0	1 (2.5)	4 (9.8)	2 (5.0)	6 (8.0)	13 (6.5)
Neurological failure	0	1 (2.5)	1 (2.4)	2 (5.0)	3 (4.0)	7 (3.5)
Other	0	0	1 (2.4)	3 (7.5)	3 (4.0)	7 (3.5)
Bacterial coinfection at admission	2 (40)	7 (18)	9 (22)	14 (35)	16 (21)	48 (24)
SAPS II	48 ± 16	43 ± 19	44 ± 21	46 ± 16	43 ± 15	44 ± 17
SOFA score	11 ± 3.7	8.0 ± 4.3	8.9 ± 5.1	10 ± 4.0*	6.8 ± 3.7	8.3 ± 4.4
*Evolution*						
ARDS	5 (100)	25 (63)	24 (59)	25 (63)	34 (45)	113 (56)
Mechanical ventilation	5 (100)	35 (88)	37 (90)	35 (88)	67 (89)	179 (89)
ECMO	0	5 (13)	3 (7.3)	0	0	8 (4.0)
VAP	1 (20)	12 (30)	8 (20)	13 (33)	17 (23)	51 (25)
***Length of stay***						
ICU (days)	21 ± 29	22 ± 29	16 ± 24	17 ± 18	17 ± 22	18 ± 23
Hospital (days)	32 ± 36	33 ± 30	34 ± 43	27 ± 22	39 ± 35	34 ± 34
***Mortality***						
Day 28	3 (60)	10 (25)	6 (15)	8 (20)	10 (13)	37 (18)
ICU	3 (60)	13 (33)	7 (17)	9 (23)	11 (15)	43 (21)
In-hospital	3 (60)	13 (33)	8 (20)	13 (33)	16 (21)	53 (26)

Data are number (%) or mean ± standard deviation, as appropriate.

ICU: Intensive Care Unit; SAPS II: Simplified Acute Physiology Score II; ARDS: Acute Respiratory Distress Syndrome; ECMO: Extra-Corporeal Membrane Oxygenation; SOFA: Sepsis-Related Organ Failure Assessment; VAP : Ventilator-Associated Pneumonia.

* *p* < 0.05 versus 2009.

In agreement with the French influenza surveillance system, a wide majority of influenza infections were caused by type A influenza virus (171/201, 85%), with half of all patients infected by A(H1N1)pdm subtype (Table 2). As shown in Table 3, patients infected with A(H1N1)pdm virus, in comparison with those infected by other virus subtypes, were more often males (sex-ratio 1.6 versus 0.8, respectively) and a younger population. These two subgroups of patients didn’t significantly differ according to the severity of the illness (Table 3) or the use of organ supports (data not shown).

**Table 2:** Virological data.

	2008	2009	2010	2011	2012	Total
***Studied catchment population***	n = 5	n = 40	n = 41	n = 40	n = 75	n = 201
***Type A***	4 (80)	40 (100)	37 (90)	40 (100) (100)	50 (67)	171 (85)
A(H1N1)pdm	0 (0)*	38 (95)	36 (88)	0 (0)*	28 (37) (37)*	102 (51)
A(H3N2)	1 (20)	0 (0)	1 (2.4)	40 (100)	22 (29)	64 (32)
A unknown subtype	3 (60)	2 (5.0)	0 (0)	0 (0)	0 (0)	5 (2.5)
***Type B***	1 (20)	0 (0)	4 (10)	0 (0)	25 (33)	30 (15)
***French data***^†^	n = 1564	n = 3171	n = 2007	n = 1417	n = 2434	n = 10,593
***Type A***	1336 (85)	3032 (96)	1044 (52)	1370 (97)	1189 (49)	7971 (75)
A(H1N1)pdm	0 (0)*	2896 (91)	835 (42)*	59 (4.2)*	581 (24)*	4371 (41)
A(H3N2)	968 (62)	0 (0)	103 (5.1)	1270 (90)	536 (22)	2877 (27)
A unknown subtype	368 (24)	136 (4.3)	106 (5.3)	41 (2.9)	72 (3.0)	723 (6.9)
***Type B***	228 (15)	139 (4.4)	963 (48)	47 (3.3)	1245 (51)	2622 (25)

Data are number (%).

*p < 0.05 versus 2009.

^**†**^From the French countrywide Influenza sentinel network *"Groupes Régionnaux d’Observation de la Grippe” (GROG;* www.grog.org).

**Table 3:** Clinical data and outcomes according to virus subtype.

	A(H1N1)pdm	Other virus subtypes				p*
	n = 102	All n = 99	A(H3N2) n = 64	B n = 30	ND n = 5	
***Demographics***						
Age (years)	57 ± 15	69 ± 15	70 ± 14	66 ± 16	64 ± 23	< 0.001
Male sex	63 (62)	44 (45)	32 (50)	12 (40) ((4(40%)	0 (0)	0.01
***Severity of illness***						
SAPS II	43 ± 18	45 ± 17	43 ± 15	49 ± 16	38 ± 16	0.48
SOFA	8.2 ± 4.7	8.3 ± 4.0	7.4 ± 4.3	6.6 ± 3.7	8.4 ± 2.6	0.86
ARDS	60 (59)	53 (54)	36 (56)	12 (40)	5 (100)	0.45
***In-hospital outcomes***						
Length of stay	38 ± 39	31 ± 26	30 ± 23	33 ± 34	28 ± 14	0.36
Mortality	27 (27)	26 (26)	15 (23)	9 (30)	2 (29)	0.97

Data are number (%) or mean ± standard deviation, as appropriate.

ND: Not documented; SAPS II: Simplified Acute Physiology Score II; SOFA: Sepsis-Related Organ Failure Assessment; ARDS: Acute Respiratory Distress Syndrome.

*A(H1N1)pdm versus all other virus subtypes.

Outcomes are presented over time (Table 1), as well as according to virus subtype (Table 3). In total, 148 (74%) patients were discharged alive from hospital. Both mortality rates and lengths of stay were not significantly different when 2009 A(H1N1) pandemic was compared to other influenza seasons (Table 1). Also, there was no influence of virus subtype regarding in-hospital outcomes (Table 3).

**Figure 1:**
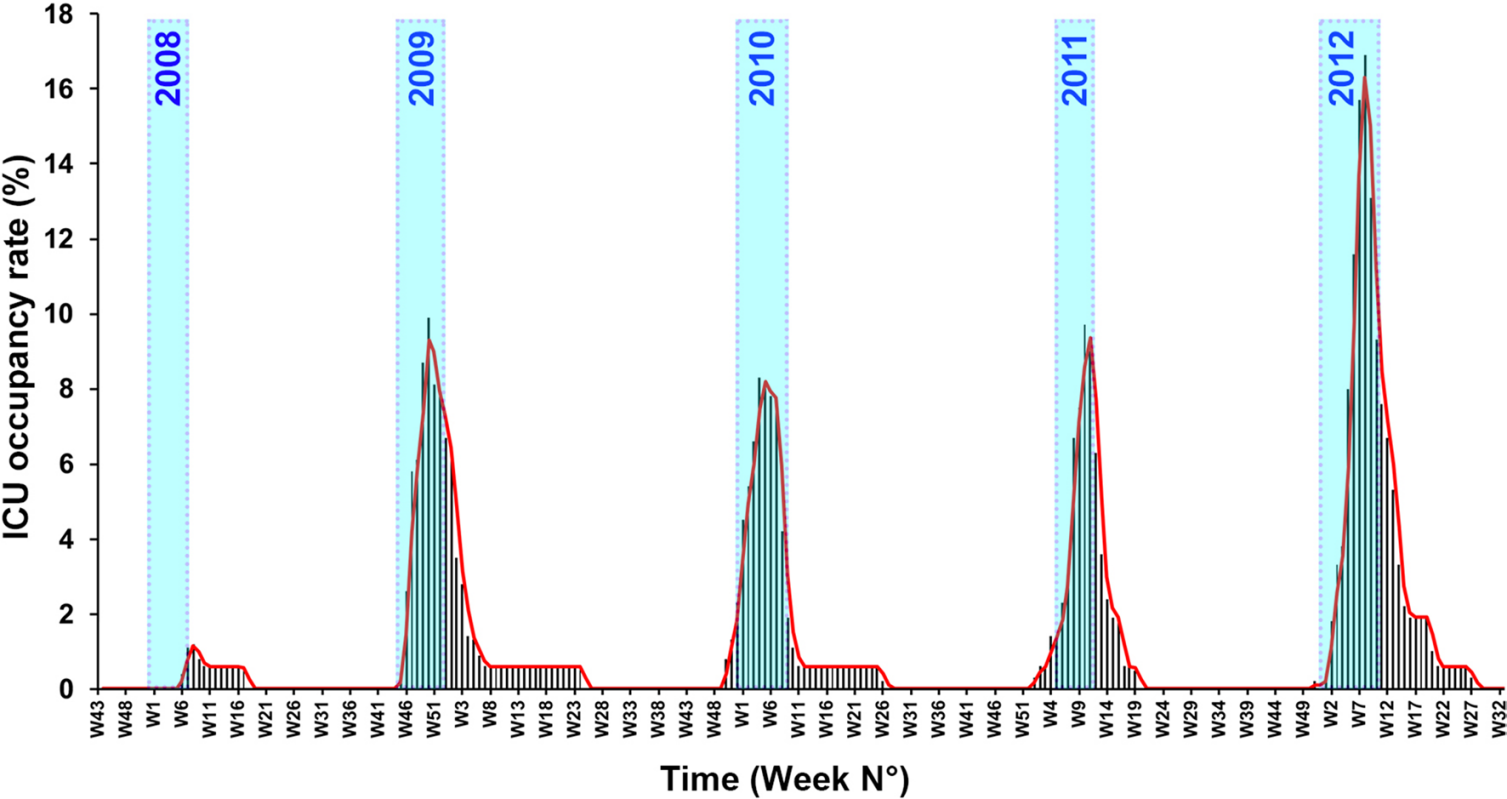
Weekly rate of ICU bed occupancy by Influenza-infected patients. At the peak of ICU activity, bed occupancy by Influenza-infected patients stayed below 10% during each epidemic period (blue bar), except during 2012 winter.

Throughout the study period, overall incidence of influenza-related ICU admission averaged 3.1 cases per 100,000 person-years. The curve of weekly ICU bed-occupancy by influenza-infected patients almost matched with the epidemic periods (Fig. 1). ICU bed-occupancy rate for these patients averaged 4.3% over the five influenza seasons. The highest mean occupancy rate (16.9%), observed during 2012 winter, was significantly higher than those noticed during previous epidemics (*p* < 0.05). Interestingly, for the 2009 A(H1N1) pandemic, the peak of ICU bed-occupancy didn’t exceed 9.9%. Of note, from 2008 to 2012, the weekly peak incidence (per 100,000 inhabitants) of emergency room visits for influenza-like illness in France was 615, 868, 754, 452 and 770, respectively (www.sentiweb.fr). When regarding factors influencing in-hospital length of stay (Table 4), only the SOFA score was independently associated with a prolonged hospitalization (OR per point, 1.12; 95% CI, 1.04–1.29; *p* = 0.005).

**Table 4:** Patients’ characteristics according to in-hospital length of stay.

	Total n = 201	≤ 24 days n = 102	> 24 days n = 99	Univariate analysis *p*	Multivariate anlaysis	
					OR (95% CI)	*p*
Age (years)	63 ± 16	64 ± 17	62 ± 15	0.18	0.99 (0.97–1.01)	0.31
Male sex	107 (53)	55 (54)	52 (53)	0.82	0.91 (0.50–1.65)	0.75
BMI (kg/m^2^)	27 ± 7.2	27 ± 7.6	27 ± 6.8	0.83		
No comorbidity	19 (9.4)	7 (6.9)	12 (12	0.20		
Immune depression	47 (23)	23 (23)	24 (24)	0.78		
Chronic pulmonary disease	71 (35	43 (42)	28 (28)	0.04	0.79 (0.41–1.53)	0.49
Chronic heart disease	42 (21)	22 (22)	20 (20)	0.81		
Symptom duration before ICU (days)	5.1 ± 5.0	4.6 ± 4.1	5.7 ± 5.7	0.36		
*Viral subtype*				0.30		
Influenza A(H1N1)pdm	102 (51)	52 (51)	50 (51)	–	0.82 (0.43–1.54)	0.53
Other Type A	69 (34)	31 (30)	38 (38)	–		
Type B	30 (15)	19 (19)	11 (11)	–		
Bacterial coinfection at admission	48 (24)	19 (19)	29 (29)	0.09	1.40 (0.68–2.86)	0.36
SAPS II	44 ± 17	45 ± 19	43 ± 15	0.70		
SOFA score at admission	8.0 ± 4.2	6.9 ± 4.0	9.1 ± 4.1	0.001	1.12 (1.04–1.29)	0.005

Data are number (%) or mean ± standard deviation, as appropriate.

BMI: Body Mass Index; ICU: Intensive Care Unit; SAPS II: Simplified Acute Physiology Score II; SOFA: Sepsis-Related Organ Failure Assessment.

## Discussion

The present study, conducted over five recent influenza seasons in a specific territory, reports the reality of ICU exposition to influenza disease. Our data emphasize the gravity of severe forms of the disease, responsible for a sustained activity in ICUs during epidemic periods. Organ failures, but not A(H1N1)pdm virus, appear to be major determinants of ICU burden related to influenza.

Influenza is the most common cause of acute infectious respiratory illness affecting between 2 and 3 million people worldwide each year^2^. Influenza-associated critical illness hospitalizations (between 5 and 19% of all hospitalizations for flu) have significantly increased over the past decades, including the 2009 A(H1N1) pandemic^3,4^. Since this pandemic, the reality for patients with severe forms of influenza remains poorly documented. Thus, we designed the present study to provide current information on influenza disease in ICUs in the real-life setting of a specific territory. The incidence for influenza-associated hospitalizations in ICUs observed over a five-year period (3.1 per 100,000 person-years) was of the same magnitude as those also exhaustively reported in Australia and New Zealand in 2009 and 2010: 3.5 and 1.1 admissions in ICU per 100,000 persons-years, respectively ^13^. ICU bed-occupancy rate by Influenza-infected patients is another parameter of interest in our comprehensive study, with a mean occupancy rate of 4.3% over the study period. The peak of ICU activity in 2009 winter only reached 9.9%. This proportion is well under the 15% critical threshold sometimes used to consider modifications in hospital admission policy, bed availability and/or cancellation of scheduled surgical activities^18^.

The occurrence of the 2009 A(H1N1) pandemic has turned the spotlight on the influenza disease. In response to the high level of media attention attracted by this pandemic, medical attitudes and practices towards influenza virus infections have changed^12^. This work is in line with this renewed interest for influenza and gives a snapshot of the influenza disease in ICUs by providing data before, during and after the pandemic. With so few influenza-infected patients during the 2008 winter, our study confirms the unrecognized burden of influenza-associated critical illness before the pandemic. Rapid influenza testing, such as RT-PCR, now helps physicians to reduce the number of undiagnosed forms of the disease^19^. Strikingly, our longitudinal study gives an original view on the last pandemic, which did not appear to be any different from other influenza seasons with regards to ICU activity, patient characteristics (except for age) and illness severity and outcomes. Only a few studies have previously compared the 2009 pandemic to other seasonal outbreaks^13,20–23^. Among them, the only one conducted in ICUs from Australia and New Zealand was coherent with our results, with no difference in outcome when the 2009 pandemic was compared to the 2010 influenza season^13^. With regards to the influence of virus type, our study does not confirm the negative impact of A(H1N1)pdm virus subtype on patients’ outcomes or specific workload in ICU. This results is in apparent discrepancy with reports that showed an association between A(H1N1)pdm infections and an increased risk of complications or deaths^20,21,23,24^. This finding might be partly explained by a lowered virulence of the subtype in the years following the pandemic^25^. In summary, the present study supports the idea of a relatively mild pandemic, with limited impact on influenza-related ICU activity.

Our study does present some limitations. First, data were only prospectively collected starting from the 2009 pandemic. We, however, chose to include data from the winter of 2008 in order to highlight the enhanced awareness towards influenza since the 2009 A(H1N1) pandemic. Second, the comprehensiveness of our work could be argued. Indeed, it cannot be excluded that some influenza-infected patients were not accounted for during inter-epidemic seasons. Nevertheless, it is acknowledged that the number of these patients is probably negligible during these periods^26^. Third, our work focused on the short-term burden of critically ill influenza-infected patients although it is well established that such severe patients require resources over a prolonged period, which exceed initial hospitalization. Thus, further studies are needed to determinate the long-term workload of critically-ill influenza infected patients, especially among elderly people. Also, the too small samples’ size to perform multivariable analyses with mortality as outcome remains a lack in the study. Finally, our results only reflect the reality of influenza-associated critical burden in the Lyon catchment area; any transposition to another territory remains uncertain. In this way, the relatively small size of the study may question the representativeness of the sample and whether the study was sufficiently powered to detect meaningful differences between influenza seasons. Larger national or international data analyses are probably needed to definitely address this question.

## Conclusions

This real-life study focuses on influenza-associated critical illness over five seasons. In a French catchment population, the 2009 A(H1N1) pandemic does not appear to have a greater influence on influenza-related ICU activity and patients’ outcomes than subsequent seasons. Since the last pandemic, ICU bed-occupancy by influenza-infected patients during outbreaks have remained high, most likely due to an enhanced awareness towards influenza disease. Severity of the disease, but not A(H1N1)pdm virus, seems to have a key role in the ICU burden related to influenza.

## Data Availability

A limited de-identified dataset is available from the corresponding author on reasonable request.
